# Modulation of the Human T Cell Response by a Novel Non-Mitogenic Anti-CD3 Antibody

**DOI:** 10.1371/journal.pone.0094324

**Published:** 2014-04-07

**Authors:** Hirokazu Shiheido, Chen Chen, Masaki Hikida, Takeshi Watanabe, Jun Shimizu

**Affiliations:** Center for Innovation in Immunoregulative Technology and Therapeutics, Graduate School of Medicine, Kyoto University, Kyoto, Japan; Federal University of São Paulo, Brazil

## Abstract

The agonistic anti-human CD3ε antibody (Ab), OKT3, has been used to control acute transplant rejection. The *in vivo* administration of OKT3 was previously shown to induce the partial depletion of T cells and unresponsiveness (anergy) in the remaining CD4^+^ T cells. However, this therapy is also associated with the systemic release of several cytokines, which leads to a series of adverse side effects. We established a novel anti-human CD3ε Ab, 20-2b2, which recognized a close, but different determinant on the CD3ε molecule from that recognized by OKT3. 20-2b2 was non-mitogenic for human CD4^+^ T cells, could inhibit the activation of T cells *in vitro*, and induced T cell anergy in *in vivo* experiments using humanized mice. Cytokine release in humanized mice induced by the administration of 20-2b2 was significantly less than that induced by OKT3. Our results indicated that the CD3ε molecule is still an attractive, effective, and useful target for the modulation of T cell responses. The establishment of other Abs that recognize CD3ε, even though the determinant recognized by those Abs may be close to or different from that recognized by OKT3, may represent a novel approach for the development of safer Ab therapies using anti-CD3 Abs, in addition to the modification of OKT3 in terms of the induction of cytokine production.

## Introduction

T cells become fully activated when they recognize an antigen and receive signals through co-stimulatory molecules. The activation of T cells is also known to be accompanied by the temporary down-modulation of the T cell receptor (TCR)/CD3 complex on the cell surface [1]–[3]. The manipulation of these events in the early stages of T cell activation, for example, by modifying antigenic determinants and/or by blocking the interaction between co-stimulatory molecules and ligands, has been shown to induce T cell unresponsiveness (anergy) [4]–[7]. We previously demonstrated that inducing the down-modulation of the TCR/CD3 complex without stimulating T cells resulted in the modulation of T cell responses [8].

In our previous study [9], we reported and characterized an Ab (Dow2) against mouse CD4^+^ T cells that was established based on its ability to induce the down-modulation of the TCR/CD3 complex and simultaneously not stimulate CD4^+^ T cells. Dow2 (rat IgG_2a_) recognized mouse CD3ε, induced T cell anergy *in vivo*, and was more effective than the well-known agonistic anti-mouse CD3ε Ab, 145-2C11, in terms of the induction of an immunosuppressive state. Although undesired cytokine production needs to be considered when the anti-CD3 Ab is used *in vivo*, Dow2 was significantly less effective at inducing cytokine production *in vivo* than 145-2C11 [9].

In the present study, we attempted to establish an anti-human monoclonal Ab that could induce the down-modulation of the TCR/CD3 complex, but not the activation of T cells as well as the anti-mouse Ab, Dow2, as described above. 20-2b2, the Ab established based on these criteria, was characterized in *in vitro* experiments and *in vivo* systems using humanized mice. 20-2b2 also recognized human CD3ε. However, the mode of recognition by 20-2b2 differed from that of the well-studied agonistic anti-human CD3ε Ab, OKT3. 20-2b2 could induce human CD4^+^ T cell anergy *in vivo* and was significantly less harmful in terms of cytokine induction *in vivo*.

## Materials and Methods

### Antibodies and reagents

Antibodies against CD3ε (OKT3 and M-20), TCRVβ8, rat IgG, and mouse IgG were purchased from eBiosciences, Santa Cruz, and BD Biosciences. Phytohemagglutinin (PHA) and pokeweed mitogen (PWM) were purchased from Sigma. All FACS data were acquired on an FACSCanto II flow cytometer (BD Biosciences) using FACSDiva software. Data were analyzed using Flowjo software (Treestar). We analyzed cytokine contents using a Bio-Plex kit (Bio-Rad) following the manufacturer's instructions.

### Ethics statement

The study protocol was approved by the Review Board for human studies in Kyoto University. Peripheral blood was obtained from consenting healthy adult donors. All donors provided written informed consent. All mice were maintained in a specific pathogen-free animal facility. The experimental procedures and housing conditions for animals were approved by the Animal Experimental Committee at Kyoto University School of Medicine, and all animals were cared for and treated humanely in accordance with the Institutional Guidelines for Experiments using Animals.

### Cell preparation

Human peripheral blood mononuclear cells (PBMCs) were isolated from the blood using a Ficoll-Paque PLUS (GE Amersham) gradient. CD4^+^ T cells were isolated using magnetic beads conjugated with the anti-CD4 Ab and a magnetic column (Miltenyi Biotec). Jurkat cells [10] and J.RT3-T3.5 [11], [12] were purchased from the American Type Culture Collection (ATCC).

### Preparation of monoclonal antibodies

Wistar rats (2 months old, purchased from Japan SLC) were intraperitoneally immunized three times every 2 wks with CD4^+^ T cells (2×10^6^) prepared from fresh healthy human PBMC, and were intravenously injected with human CD4^+^ T cells (1×10^7^) one month later. Spleen cells were fused with P3X63Ag8.653 myeloma cells (from ATCC) 3 days after the final immunization. Supernatants (SNs) from the resulting hybridomas were screened for their ability to induce the down-modulation of the TCR/CD3 complex. Selected hybridomas in terms of stable-inducing activity were subjected to subsequent cloning. One clone, 20-2b2 (rat IgG_2a_), was established.

### Cell culture

Human T cells (1×10^4^/w) were stimulated with anti-CD3/CD28-coated beads (2×10^4^/w, Dynabeads, Invitrogen). Cells were maintained at 37°C with 5% CO_2_ in RPMI supplemented with 10% fetal bovine serum, penicillin (100 U/ml), and streptomycin (0.1 mg/ml) unless otherwise stated. The proliferation of T cells was assessed by measuring the incorporation of [^3^H]TdR (37 kBq/well) for the final 4 hr of a 2- to 3-day culture.

### Immunoblotting

Cells were washed twice with PBS, and lysed for 1 hr at 4°C in lysis buffer (50 mM Tris-HCl, pH 7.5, 150 mM NaCl, 5 mM EDTA, and 1% Triton X-100) supplemented with protease and phosphatase inhibitors (both purchased from Nacalai Tesque, Japan). Cell lysates were then separated from the debris by centrifugation at 20,000 *g* for 15 min at 4°C. Protein concentrations were determined by the BCA protein assay (Thermo), 5–20 μg cell lysates were separated by SDS-PAGE under reduced conditions, and proteins were electrotransferred onto PVDF membranes (Millipore). After blocking with blocking-one (Nacalai Tesque) in Tris-Buffered saline containing 0.1% Tween 20, the membrane was incubated overnight at 4°C with the indicated primary antibody, washed, and subjected to chemiluminescence detection with the HRP-conjugated secondary antibody with ECL (Millipore). In some experiments, cell lysates (500–1000 μg) were incubated with the indicated primary antibody for 2 hr at 4°C. Immunocomplexes were precipitated with protein A-Sepharose (Sigma) for 1 hr at 4°C. Immunoprecipitates were washed four times with ice-cold wash buffer (50 mM Tris-HCl, pH 7.5, 150 mM NaCl, and 1% Triton X-100). Immunoprecipitated proteins were eluted with sample buffer containing 100 mM DTT and heated for 10 min at 96°C.

### Plasmid preparation and transfection

Total RNA was extracted from 3×10^6^ Jurkat cells using TRIzol reagent (Invitrogen) according to the manufacturer's instructions, followed by reverse transcription with the Superscript III first-strand synthesis system for RT-PCR (Invitrogen) using oligo(dT)_20_. The resultant cDNA was used as a template for PCR using 5'-AAGCGGCCGCACCATGCAGTCGGGCACTCA-3' and 5'-AAGGATCCACCTCAGATGCGTCTCTGATTCAGG-3' as forward and reverse primers, respectively, to obtain the full-length of the human CD3ε (hCD3ε) gene. The PCR product was cloned into the NotI/BamHI site of the pQCXIX-derived pQCXIXGFP vector that encoded the GFP gene downstream of the IRES site, resulting in the pQC-hCD3εGFP expression vector. We transfected Yac-1 cells with the human CD3ε expression vector, pQC-hCD3εGFP, using Lipofectamine LTX and Plus Reagent (Invitrogen) according to the manufacturer's instructions. GFP^+^ cells were sorted and expanded. This cycle was repeated, and the resulting stable line was used as hCD3ε-Yac-1 cells.

### Humanized mice

Six-week-old female NOD/shi-scid/γc^null^ (NOG) mice were obtained from the Central Institute for Experimental Animals (CIEA). Mice were irradiated with 2 Gy, and 1×10^5^ cord blood CD34^+^ cells, which were obtained from Lonza Walkersville Inc., were transferred into these mice by an intravenous injection. Mice were then used as humanized NOG (Hu-NOG) mice 20–30 weeks later.

## Results

### 20-2b2 inhibited CD4^+^ T cell activation

**Figure 1:**
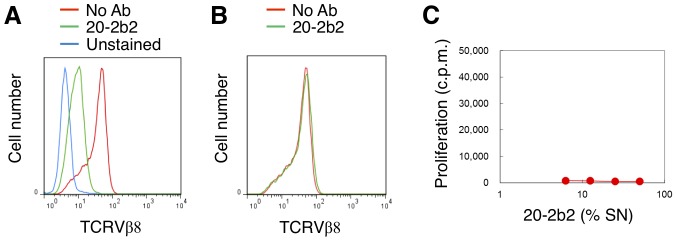
Non-stimulatory 20-2b2 induced the down-modulation of TCR expression. (A) Jurkat cells were cultured in the presence (green) or absence (red) of 20-2b2 (50% SN) at 37°C. Cells were washed and stained with the anti-TCRVβ8 Ab two hours later. Unstained Jurkat cells are shown as a blue line. (B) Jurkat cells were cultured in the presence (green) or absence (red) of 20-2b2 (50% SN) at 4°C for 20 min, and then stained with anti-TCRVβ8 Ab conjugated with FITC. (C) The PBMCs of healthy donors were cultured with the titrated amount of 20-2b2. Cell proliferation was measured two days later. Data are expressed as the mean ± SD of triplicate cultures. The proliferation of PBMCs without a stimulus was measured as 718±346 cpm. Results are representative of three independent experiments (A–C).

Rats were immunized with human CD4^+^ T cells prepared from PBMCs, spleen cells from immunized rats were fused with myeloma cells, and the resulting hybridomas were selected based on their ability to induce the down-modulation of the TCR/CD3 complex in the human T cell line, Jurkat, which used TCRVβ8 [13]. The selected hybridomas were further screened for their ability to have no stimulatory activity on T cells in PBMCs. We established the monoclonal Ab, 20-2b2 (rat IgG_2a_) from this two step screening. As shown in Figure 1A, pre-treating Jurkat cells with 20-2b2 at 37°C for two hours resulted in diminished staining with the anti-TCRVβ8 Ab. In contrast, pre-treating Jurkat cells with 20-2b2 at 4°C had no influence on staining with the anti-TCRVβ8 Ab (Figure 1B), which demonstrated that neither 20-2b2 nor the anti-TCRVβ8 Ab were competitive in staining (Figure 1B), and that pre-treating Jurkat cells with 20-2b2 at 37°C resulted in the down-modulation of TCR (Figure 1A). Whole PBMCs were then cultured with a titrated amount of 20-2b2. However, 20-2b2 did not induce any proliferation (Figure 1C). PBMCs cultured with an agonistic anti-CD3 Ab (OKT3 at 1 ng/ml) as a positive control led to the marked proliferation (36,602±2,228 cpm in the same experiment shown as Figure 1C) and production of IL-2 (16 U/ml). In contrast, stimulating PBMCs with 20-2b2 resulted in less than 1 U/ml of IL-2 being produced. These results demonstrated that 20-2b2 had the ability to induce the down-modulation of the CD3/TCR complex (Figure 1A) without activating T cells (Figure 1C).

**Figure 2:**
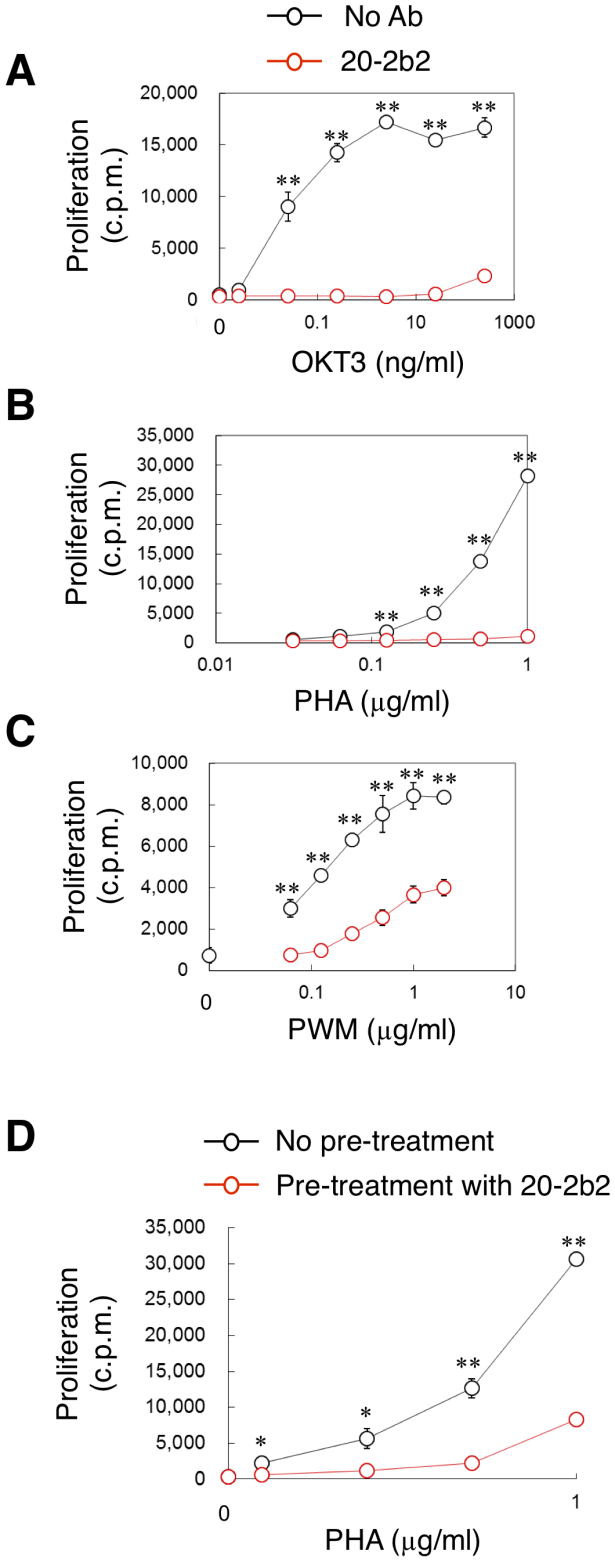
20-2b2 inhibited the activation of T cells *in vitro*. (A–C) The PBMCs of healthy donors were cultured with an anti-CD3 Ab (OKT3) (A), PHA (B), or PWM (C) in the presence (red symbols) or absence (black) of 20-2b2 (50% SN). (D) PBMCs were pre-treated with medium (black) or 20-2b2 (red) at 37°C. Cells were washed three times and cultured with PHA two hours later. Cell proliferation was measured two days later. Values are shown as the mean ± SD of triplicate cultures. Data are representative of more than three (A–C) and three (D) independent experiments. *p<0.01, **p<0.001 by the Student's *t*-test.

We further investigated the biological activities of 20-2b2 *in vitro*. Whole PBMCs were cultured with various stimuli (agonistic anti-CD3ε Ab, OKT3; T cell-mitogen, PHA; and T cell- and B cell-mitogen, PWM) in the presence or absence of 20-2b2. 20-2b2 strongly inhibited the *in vitro* activation of T cells against any stimuli (Figure 2A–C). Furthermore, the pre-treatment of cells with 20-2b2 resulted in the inhibition of T cell activation (Figure 2D). These results demonstrated that 20-2b2 was not only a non-stimulatory (Figure 1C), but also inhibitory Ab for T cells (Figure 2A–C), and suggested that 20-2b2 was unlikely to be toxic for T cells *in vitro* (Figure 2D).

### 20-2b2 recognized CD3ε

**Figure 3:**
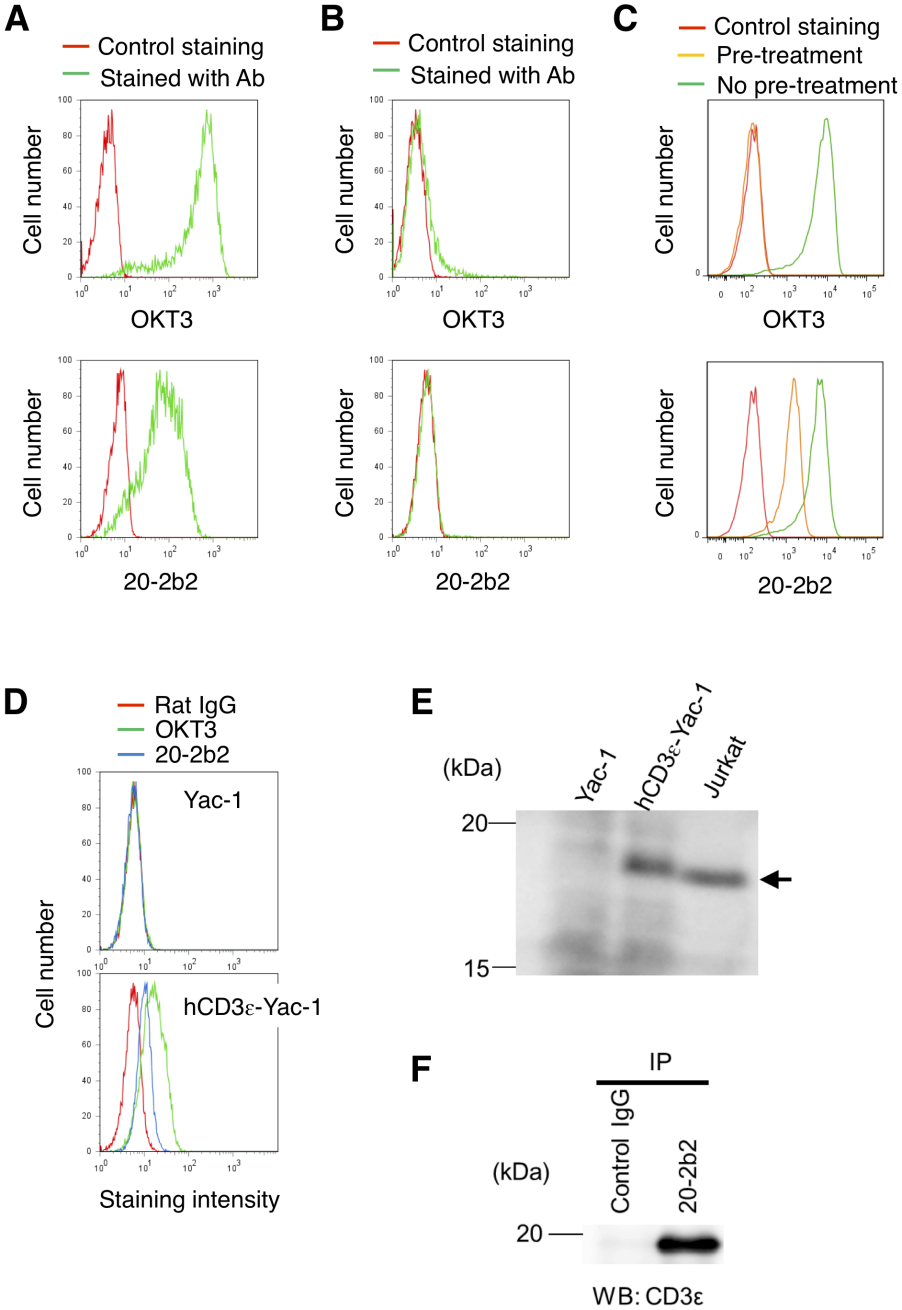
20-2b2 recognized CD3ε. (A, B) Jurkat (A) or J.RT3-T3.5 cells (B) were stained with anti-CD3ε Ab (OKT3) or 20-2b2 (green lines). Unstained cells are shown as red lines. (C) Jurkat cells were pre-treated with 20-2b2 (top panel) or OKT3 (bottom panel), and were then stained with the indicated Ab (yellow lines). Jurkat cells stained without the pre-treatment are shown as green lines. Negative control staining is shown as red lines. (D) Yac-1 or Yac-1 cells transfected with human CD3ε (hCD3ε-Yac-1) were stained with the anti-CD3ε Ab (OKT3) (green) or 20-2b2 (blue). Cells stained with rat IgG as a negative control are shown as red lines. (E) Cell lysates prepared from the indicated cells were separated in SDS-PAGE and blotted with 20-2b2. Arrow: the target molecule recognized by 20-2b2. (F) The lysate from Jurkat cells was immunoprecipitated (IP) with 20-2b2 or control rat IgG. Immunoprecipitates were separated by SDS-PAGE. The blot was probed (WB) with the anti-CD3ε Ab (M-20). Results are representative of three independent experiments (A-F).

We then attempted to identify the target molecule recognized by 20-2b2. 20-2b2 could stain Jurkat cells as well as the anti-human CD3ε Ab, OKT3 (Figure 3A). On the other hand, the mutant cell line derived from Jurkat cells, J.RT-3-T3.5 [11], [12], which do not express either CD3 or TCR on their surface, was not stained with OKT3 or 20-2b2 (Figure 3B). Furthermore, the pre-treatment of Jurkat cells with 20-2b2 resulted in unstaining with OKT3 (Figure 3C, top panel), while pre-treating with OKT3 led to diminished staining with 20-2b2 (Figure 3C, bottom panel). Taken together, these results prompted us to assume that 20-2b2 may recognize a component of the CD3/TCR complex, possibly the determinant on CD3ε molecules. To confirm this, the mouse T cell line, Yac-1, was transfected with human CD3ε (hCD3ε), and a stable line was established (see Materials and Methods). Mouse CD3ε in Yac-1 cells was expected to be partly substituted by hCD3ε in the resulting transfected stable line (hCD3ε-Yac-1). The anti-hCD3ε Ab (OKT3) could stain hCD3ε-Yac-1 (Figure 3D). 20-2b2 could also stain hCD3ε-Yac-1, but with less intensity. We performed more biochemical experiments. Lysates prepared from Yac-1, hCD3ε-Yac-1, and Jurkat cells were subjected to SDS-PAGE under non-reducing conditions, transferred onto a membrane, and blotted with 20-2b2 (Figure 3E). The resulting specific band with a molecular weight of 18 kDa was detected in both lysates from the hCD3ε-positive cells, hCD3ε-Yac-1, and Jurkat cells, but not from the hCD3ε-negative cells, Yac-1. Moreover, we confirmed that the immunoprecipitated protein from Jurkat cell lysates with 20-2b2 was detected with the anti-CD3ε Ab (Figure 3F). Taken together, these results demonstrated that 20-2b2 recognized human CD3ε, whereas the determinant recognized by 20-2b2 may be close to, but different from that recognized by OKT3 (Figure 3C). In addition, 20-2b2 may have lower affinity against CD3ε molecules than that of OKT3 because weaker staining with 20-2b2 was observed, as shown in Figure 3A and D.

### 20-2b2 induced CD4^+^ T cell anergy *in vivo*

**Figure 4:**
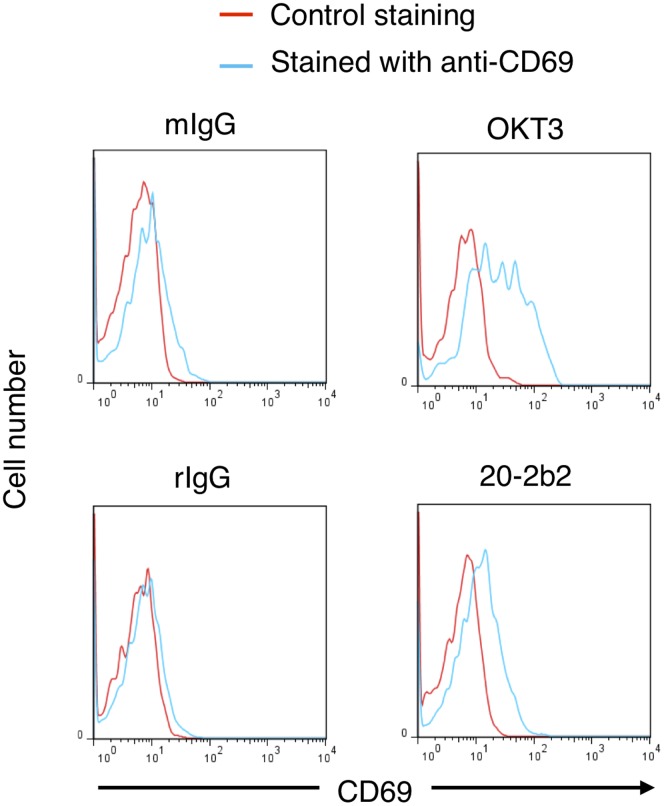
20-2b2 induced the expression of CD69 on CD4^+^ T cells *in vivo*. Hu-NOG mice were intravenously inoculated with one of the following Abs (20 μg/mouse); mouse IgG_2a_ (mIgG), rat IgG (rIgG), OKT3, or 20-2b2. Spleen cells were stained with the anti-CD4 Ab and a control Ab or anti-CD69 Ab six hours later. Cells stained with a control Ab (red) or anti-CD69 Ab (blue) in CD4^+^ T cells were shown as a histogram. Results are representative of three individual Hu-NOG mice.

The *in vivo* biological activities of 20-2b2 were then examined. 20-2b2 (20 μg/mouse) was intravenously injected into humanized NOG (Hu-NOG) mice (Figure 4). We observed that almost all CD4^+^ T cells in the peripheral blood were depleted 6 hr after the injection of 20-2b2 (% CD4^+^ T cells in the peripheral blood at 0 hr: mean ± SD = 17.0±3.0, n = 3; at 6 hr: 1.2±0.3, n = 3; *p*<0.001 by the Student's *t*-test). We also obtained similar results by injecting the agonistic anti-CD3 Ab (OKT3, 20 μg/mouse, % CD4^+^ T cells in the peripheral blood: 15.3% at 0 hr to 1.2% at 6 hr in a representative experiment). In contrast, a significant number of CD4^+^ T cells existed in the spleen at the same time point. Furthermore, the slightly increased expression of CD69 (the earliest inducible activation marker of T cells) was observed on CD4^+^ T cells from 20-2b2-injected Hu-NOG mice, although this expression level was lower than that induced by OKT3 (Figure 4, the increase in staining  =  (the mean fluorescence intensity (MFI) by anti-CD69 Ab staining) – (MFI by control staining): mean ± SD = 22.6±8.38 in OKT3-injected Hu-NOG mice, n = 3; 10.4±4.84 in 20-2b2-injected Hu-NOG mice, n = 3; *p*<0.05 by the Student's *t*-test). These results demonstrated that 20-2b2 was partly depletive *in vivo* and actively functioned against CD4^+^ T cells *in vivo*.

**Figure 5:**
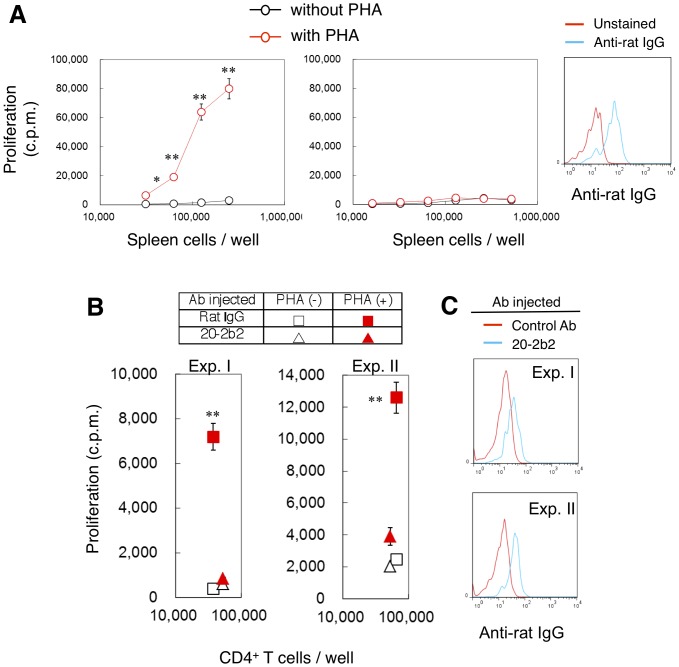
20-2b2 induced CD4^+^ T cell anergy *in vivo*. (A) Hu-NOG mice were intravenously injected with one of the following Abs (100 μg/mouse); control Ab (rat IgG) (left panel) or 20-2b2 (middle panel). Whole spleen cells were prepared and cultured in the presence (red) or absence (black) of PHA (1 μg/ml) twenty-four hours later. Cell proliferation was measured two days later. Values are shown as means ± SD of triplicate cultures. Aliquots of spleen cells from 20-2b2-injected Hu-NOG mice were stained with the anti-CD4 Ab with or without the anti-rat IgG Ab. Staining with the anti-rat IgG Ab on CD4^+^ T cells is shown as blue line. CD4^+^ T cells without anti-rat IgG Ab staining are shown as red lines. Data are representative of more than five experiments. (B) Two NOG mice (2 Gy irradiated) as recipients were inoculated with spleen cells from one Hu-NOG mouse. One NOG mouse as a recipient was injected with the control Ab (square, 20 μg/mouse) and another NOG mouse with 20-2b2 (triangle, 20 μg/mouse). CD4^+^ T cells were prepared from these NOG mice seven days later and cultured in the presence (closed red) or absence (open black) of PHA (1 μg/ml). Cell proliferation was measured three days later. Values are shown as the mean ± SD of triplicate cultures. (C) Aliquots of CD4^+^ T cells from control Ab-injected (red lines) or 20-2b2-injected NOG mice (blue lines) in (B) were stained with anti-rat IgG. Data from two individual experiments (Exp. I and Exp. II) are shown. *p<0.01, **p<0.001 by the Student's *t*-test, cell proliferation induced by the PHA stimulation with the control Ab versus the 20-2b2 injection.

Whole spleen cells were prepared 24 hr after Hu-NOG mice were inoculated with 20-2b2. Spleen cells from control Ab-injected Hu-NOG mice mounted cell proliferation against the T cell mitogen, PHA (Figure 5A, left panel). On the other hand, cell proliferation was not observed in spleen cells from 20-2b2-injected Hu-NOG mice (Figure 5A, middle panel); we confirmed the existence of T cells in these spleen cells (for example, in the same experiment, as shown in Figure 5A middle panel, 32.3% CD4^+^ T cells and 16.9% CD8^+^ T cells were contained in human CD45^+^ cells). We also observed the staining of CD4^+^ T cells from 20-2b2-injected Hu-NOG mice with the anti-rat IgG Ab (Figure 5A, right panel), which indicated that the injected 20-2b2 was binding and remained on the T cell surface.

We further investigated the *in vivo* function of 20-2b2. Spleen cells from unmanipulated Hu-NOG mice were transferred into two other NOG mice on day 0, which were then injected with the control Ab or 20-2b2 on the same day. CD4^+^ T cells (>80% purity) were prepared from these mice on day 7, and cultured with PHA for three days. On day 7 after the 20-2b2 injection, we still observed the low intensity staining of CD4^+^ T cells from 20-2b2-injected NOG mice with the anti-rat IgG Ab (Figure 5C), which indicated that injected 20-2b2 still slightly remained on the cell surface. As shown in Figure 5B, whereas CD4^+^ T cells from control Ab-injected mice could proliferate following the PHA stimulation, CD4^+^ T cells from 20-2b2-injected mice exhibited T cell anergy. Taken together, these results indicated that 20-2b2 possessed the ability to induce anergy in T cells *in vivo*. Furthermore, the injection of 20-2b2 alone without the simultaneous injection of any antigen resulted in the induction of T cell anergy, which demonstrated that the induction of T cell anergy by 20-2b2 did not require an antigen stimulation.

**Figure 6:**
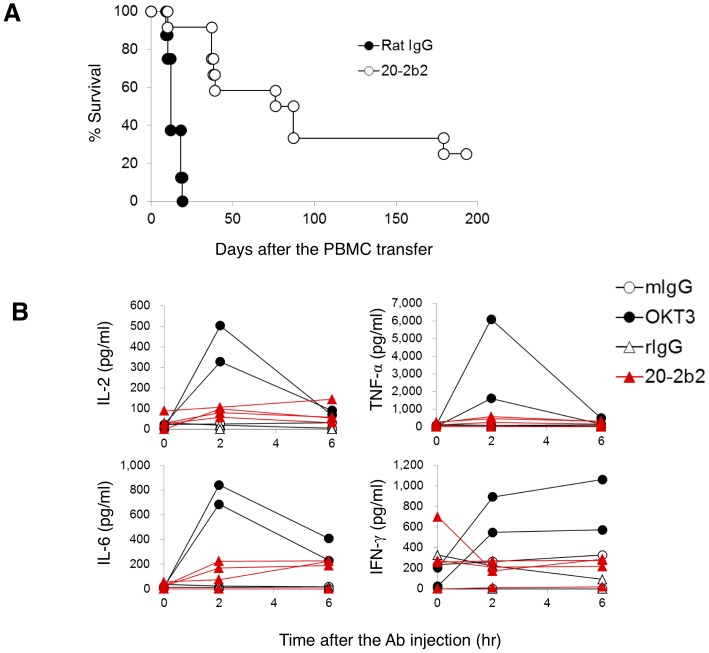
Immunosuppressive activity of 20-2b2 *in vivo*. (A) Irradiated (2 Gy) NOG mice were intravenously injected with whole PBMCs (2×10^6^/mouse) from a healthy donor on day 0. Mice were intravenously injected with the control Ab (closed symbols, n = 8) or 20-2b2 (open symbols, n = 12) on days 2, 9, and 16 (20 μg/mouse/injection). The survival of mice was monitored. Data from two independent experiments were pooled. *p*<0.001 in rat IgG versus 20-2b2, by the F-test. (B) Hu-NOG mice were intravenously injected with one of the following Abs (20 μg/mouse); control mouse IgG_2a_ (mIgG), rat IgG (rIgG), OKT3, or 20-2b2. Serum samples were obtained 0, 2, and 6 hours after the injection. Cytokine levels were measured. Data from the same Hu-NOG mouse are linked with a line. Data from two experiments are shown. Standard deviations were less than 10% of the mean.

We used a xenogenic graft versus host disease (GVHD) model to examine the ability of 20-2b2 to suppress immune responses *in vivo* (Figure 6A). Irradiated NOG mice were transferred with PBMCs from a healthy donor on day 0 and then injected with a control Ab or 20-2b2 on days 2, 9, and 16. All NOG mice injected with the control Ab died within 19 days (median graft survival time (MST) = 13.75 days). In contrast, 20-2b2 significantly prolonged the survival of recipient mice (MST = 51.38 days). We examined whether human CD4^+^ T cells existed in the peripheral blood of NOG mice transferred with human PBMCs and treated with 20-2b2. In a representative experiment, CD4^+^ T cells ranging from 12.5 to 34.9% were detected on day 29 in the peripheral blood of NOG mice (n = 5) treated with 20-2b2. On day 40, 85-89% of CD4^+^ T cells were observed. Taken together, these results demonstrated that the inoculation with 20-2b2 induced a temporary decrease in CD4^+^ T cells in the peripheral blood, as described above, that 20-2b2 was a strong immunosuppressive agent against human cells even *in vivo*, and that this prolongation was not simply due to the depletion of T cells. The injection of 20-2b2 starting from day 2 was effective even after the transfer of PBMCs into NOG mice on day 0, which suggested that 20-2b2 was immunosuppressive even for primed T cells and/or T cells being primed.

The possibility of the induction of cytokine release has to be considered when the anti-CD3 Ab is used *in vivo* [14]–[16]. Therefore, Hu-NOG mice were injected with the control Abs, OKT3 or 20-2b2, and serum was collected 2 and 6 hours later. As shown in Figure 6B, OKT3 strongly induced the release of all cytokines examined. In contrast, 20-2b2 weakly induced the release of cytokines. These results demonstrated that 20-2b2 was an ideal and safer Ab in terms of cytokine release for inducing an immunosuppressive state *in vivo*.

## Discussion

The anti-human CD3ε Ab, OKT3, was the first monoclonal Ab to be used clinically [17]–[20]. OKT3 exhibited strong immunosuppressive potency in its first clinical trials. However, because of its lymphocytic mitogenic activity, OKT3 was found to be a potent inducer of cytokines *in vivo*, including IL-2, IFN-γ, and, especially, TNF-α [21], [22]. The production of these cytokines resulted in various adverse side effects [21], [23]. To solve these problems, a modification to the Fc region in the Ab and/or the humanization of Abs were introduced [24]–[27], and engineered Abs were characterized using *in vivo* models [27]–[31]. Although these Abs exhibited significantly diminished mitogenic activity (although not completely non-mitogenic), the mild release of cytokines has still been reported [28], [29], [32], [33]. These studies were performed by focusing on limited anti-CD3ε Abs, including OKT3 or other particular anti-CD3ε Abs. Abs against the same molecule, but a different determinant, are known to exhibit different biological activities and effectivities [8], [34]. Therefore, this may represent one approach to establish Abs against novel determinants on the CD3ε molecule for the development of and improvements in Ab therapies focused on the CD3ε molecule. In the case of 20-2b2 reported in this study, a modification in the Fc region of 20-2b2 may improve its less, but not null, adverse effects (Figure 6B). Furthermore, as shown in this study, establishing new Abs with the ability to down-modulate the TCR/CD3 complex, but not to stimulate T cells, that recognize the determinant on CD3ε, which is close to or different from that recognized by well-characterized anti-CD3ε Abs, such as OKT3, may provide a new tool for inducing immunosuppression.

We demonstrated that 20-2b2 was not simply a binding Ab, but actively functioned against CD4^+^ T cells *in vivo*; e.g. the induction of the activation marker, CD69 (Figure 4). However, its efficiency in inducing the expression of CD69 was lower than that of OKT3. This low efficiency or incompleteness may be important in inducing T cell anergy. Previous studies demonstrated that manipulations/modifications in the early stage of T cell activation led to the induction of T cell anergy [4], [5], [7]. Therefore, the slight induction of the activation marker by 20-2b2 was attributed to an incomplete activation process in the early stage of T cell activation, and may result in the induction of T cell anergy (Figure 5). The molecular mechanisms by which 20-2b2 induces T cell anergy need to be investigated in more detail.
